# Digital surveillance in post‐coronavirus China: A feminist view on the price we pay

**DOI:** 10.1111/gwao.12471

**Published:** 2020-06-08

**Authors:** Ai Yu

**Affiliations:** ^1^ Institute of Management Studies Goldsmiths University of London

**Keywords:** digital surveillance, COVID‐19, feminist

1

George Fu Gao, Director of the China Centre for Disease Control and Prevention (CDC), joined a live streaming panel^1^ on 25 April 2020 to share insights into the latest developments in China's response to COVID‐19. At a time when the country's lockdown measures are relaxing, he advocated ‘health code and travel cards’, amongst other measures, and emphasized that the success of biometric tools and devices depends very much on such high‐tech applications as big data and AI to carry out targeted measures, for example, to trace someone's travel history, close contacts or the transmission chain of infections, etc.

The health code and travel cards that Mr Gao referred to are essentially a colour‐coded health system that dictates where citizens can go. Indeed, as people in China resume their lives, local authorities have been trying to control the further spread of disease by controlling citizens’ movements. This is done via smartphone software installed in WeChat, used by 1.16 billion people, and Alipay, used by 900 million. The program issues everyone with a coloured health code if they fill out a quick health survey, and the code dictates whether they can leave the house or not. While western nations are critical of this mass surveillance tool and ask what else the data may be used for, scholars^2^ are more concerned about the high likelihood that this mass data collection could be accepted as the norm even after the coronavirus has become less of a threat to the public.

Philosophers^3^ share this concern. During the COVID‐19 outbreak in Europe, Giorgio Agamben stuck his neck out by criticizing governments for continuously taking advantage of biotechnological interventions to establish and extend states of exception. Jean‐Luc Nancy has rejoined that there are only exceptions these days because everything we once considered normal is now falling apart. Divya Dwivedi and Shaj Mohan, philosophers from India, remind us that there is no unique paradigm of exception in the long run, if, like viruses and human bodies, ‘everything learns to live with everything else’. They then point out that the term ‘biopolitics’ takes its stand from the assumption of nature's temporality, and thus neglects what looks like disaster from the standpoint of our interest in life and the responsibility that we assume for saving it. This raises the serious question: are we worth saving, and if so, at what cost?

In what follows, I look at this question through a Foucault‐inspired feminist lens on surveillance. I examine a case that illustrates how digital surveillance, despite being perceived as a successful development in China's response to COVID‐19, reinforces the country's gender‐biased discourses on contagious disease and sexuality. In doing so, I want to emphasize that the states of exception that governments can establish and extend are not gender‐neutral. Given the feminist interest in and responsibility for life, this fact compels us to react to states of exception from a freedom‐enabled gender perspective. It is to be hoped that this perspective would then help to promote socio‐political practices that allowed society to challenge the normalizing male gaze induced by digital surveillance.

According to Heilongjinag Provincial CDC, a confirmed case of COVID‐19 was identified in Haerbin on 9 April, thanks to ‘cluster tracing and big data comparisons’.^4^ While the patient, Mr X, showed symptoms, his son Mr A, Mr A's girlfriend Ms B, Ms B's daughter Ms C and Ms C's boyfriend Mr D were confirmed as asymptomatic cases. It turned out that Ms C might have been infected by her downstairs neighbour, a postgraduate student E, who was reading for a degree at a US university. An officer from Haerbin CDC later revealed more details in a media briefing: E flew from New York to Haerbin on 19 March 2020 and was then asked to remain self‐isolated at home for 14 days. E tested negative on both 31 March and 3 April and has led a normal life ever since.

Ms C's personal and private information, including her full address and travel history were included in Haerbin CDC's coronavirus updates and briefings,^5^ while Ms B and her boyfriend Mr A were criticized for ‘not cooperating in the investigation’ and ‘concealing information about their health status, travel history and the people who they had contacted’. They were thus believed to violate the ‘Criminal Law of the People's Republic of China’ and the ‘Law of the People's Republic of China on the Prevention and Treatment of Infectious Diseases’, and were told that ‘the police and security organizations [would] hold them legally accountable’ for their actions.

In the following days, information on Ms C — her name, age, profession, national ID number, her selfies and the video clips posted on her social media accounts — were quickly put together by netizens through the so‐called ‘cyber manhunt’ approach. When the China Central Television's ‘CCTV news’ (its official Weibo account) mentioned in a tweet that E was a man, many media articles began to speculate about the relationship between him and Ms C. This tweet was immediately reposted by other media outlets (e.g., Global Times, thepaper.cn), but has recently been deleted. In a widely circulated online article,^6^ ‘a contact map’ was drawn to show how Ms C had been infected and had then spread the virus to more than 17 people. Ms C was accused of having an affair with E behind her boyfriend's back. The sarcastic catchphrase ‘Haerbin love story’ was coined to account for the spread of the virus. At the same time, Ms B was also subjected to a ‘cyber manhunt’ and mocked for having a boyfriend at her age (54!). In a different article, Ms C was described as ‘pretty’, ‘unemployed’, but ‘rich’, because her mother owned several properties in the city. Ms B, in contrast, was described as someone who ‘gave birth to her daughter when she was only 20’.

On 15 April, the ‘Haerbin love story’ took a dramatic and unexpected turn when Haerbin CDC revealed at a press conference that E was in fact a woman. According to the health authority, Ms E and Ms C had not known each other, but had shared a communal elevator, which may have encouraged the virus to spread. The fact that they lived only one floor apart was also cited as an epidemiological link in view of the structural conditions of the apartment building where they live. After the press conference, a small handful of content producers on social media maintained that Ms C and Ms E were in a relationship, this time, as a lesbian couple. The mainstream media and major social media networks nonetheless remained collectively silent on the issues at stake: (i) why was Ms E mis‐described as a man?; (ii) why was Ms C's personal and private information revealed to the public when this kind of information had nothing to do with preventing and controlling disease?; and (iii) was there any evidence against Ms B that held her legally accountable for anything whatsoever?

The three questions above are closely related to digital surveillance. Clearly, digital surveillance is a particularly effective form of social control during a global pandemic because it takes hold of people at the level of their bodies, gestures, desires and habits to create individuals who are willing to take (or to avoid) certain subjective positions. In the case of Ms B and Ms C, they were condemned for not taking to an extreme the practices to which they would subject themselves in their efforts in a time of pandemic to comply with the norms of a fantasized feminine citizen: a self‐disciplined woman in a stable and legally recognized relationship, who would never cause the authorities any trouble. The authorities and their accomplices (netizens), on the other hand, can always exercise a male gaze over female citizens such as Ms B and Ms C. This male gaze exercises a presumed right to make normalizing judgements concerning the behaviours of women in a specific category: unmarried, unemployed, socially or sexually active, etc. These judgements are embedded in the dominant discourses on, for example, the nature of contagious disease and of sexuality.

What seems most alarming is the fact that the Chinese government and public health authorities at all levels will never ever admit that digital surveillance is somehow related to gender. This non‐relationship, however, is still a relationship, according to Foucault (1980): one of a deeper sort. For example, Ms E was taken to be a man, and this was plainly a mistake. The authorities may keep silent about it, pretending that it was an unintended error of little importance — but isn’t this ‘unintended’ slip just a sign of the collective unconscious? After all, it may well be convenient to present Ms E as a man, if we recall that digital surveillance in China presupposes a male gaze. In fact, for the sixth year in a row, China has slipped down the rankings of the World Economic Forum's^7^ global index for the gender gap. Its gender gap has widened just when other countries are closing theirs. China ranked 57^th^ in 2008 and is now 106^th^.

Indeed, when digital surveillance becomes powerful enough to extend to self‐reflection and self‐consciousness, it can serve as one of the mechanisms that link the macro‐issues of gender oppression to the micro‐level of gendered practices and relations. While the health code and travel cards alike may expand an individual citizen's capacity for self‐knowledge and self‐care in a time of COVID‐19, it nonetheless opens the door to unprecedented levels of biometric surveillance, extends the regulatory mechanisms of public health authorities, and augments the cooperation of bio‐power and patriarchal power.

If we take digital surveillance seriously from a feminist viewpoint, we perhaps would want to help women in China shed their subordinated status by practising what the feminist scholar Wendy Brown, inspired by Foucault, called the ‘politics of resistance’. By insisting that freedom needs more than being liberated from domination, Foucault points to the importance of establishing new patterns of behaviour, attitudes and cultural forms that work to empower the vulnerable and, in this way, to ensure that mutable relations of power do not congeal into states of domination. Brown (1995, p. 49) therefore argues for political practices that seek to cultivate socio‐political space in which dominant social and political norms can be questioned and the nature of the good for women can be discussed in a specific context. The creation of such democratic space for discussion will then contribute to teaching us how to have public conversations with each other and enable us to argue about a vision of the common good from our diverse perspectives rather than from some assumed common identity — a fantasized image of the female citizen — in fighting against a global pandemic.

## DECLARATION OF CONFLICTING INTERESTS

The author declared no potential conflicts of interests with respect to the authorship and/or publication of this article.

## ENDNOTES

1 https://www.youtube.com/watch?v=aIwmL2QiK7w

2 https://www.theguardian.com/commentisfree/2020/apr/18/mass‐surveillance‐coronavirus‐technology‐expansion

3 https://www.journal‐psychoanalysis.eu/coronavirus‐and‐philosophers/

4 https://www.thepaper.cn/newsDetail_forward_6984768

5 https://k.sina.cn/article_6221427801_v172d3745901900n6jv.html?from=local

6 https://m.iyxwzx.com/guonei/40633.html

7 http://www3.weforum.org/docs/WEF_GGGR_2020.pdf
